# Gene Targeting in Disease Networks

**DOI:** 10.3389/fgene.2021.649942

**Published:** 2021-04-23

**Authors:** Deborah Weighill, Marouen Ben Guebila, Kimberly Glass, John Platig, Jen Jen Yeh, John Quackenbush

**Affiliations:** ^1^Department of Biostatistics, Harvard T. H. Chan School of Public Health, Harvard University, Boston, MA, United States; ^2^Channing Division of Network Medicine, Brigham and Women’s Hospital, Boston, MA, United States; ^3^Harvard Medical School, Harvard University, Boston, MA, United States; ^4^Departments of Surgery and Pharmacology, Lineberger Comprehensive Cancer Center, University of North Carolina at Chapel Hill, Chapel Hill, NC, United States

**Keywords:** cancer genomics, network medicine, gene targeting, differential targeting, gene regulatory networks

## Abstract

Profiling of whole transcriptomes has become a cornerstone of molecular biology and an invaluable tool for the characterization of clinical phenotypes and the identification of disease subtypes. Analyses of these data are becoming ever more sophisticated as we move beyond simple comparisons to consider networks of higher-order interactions and associations. Gene regulatory networks (GRNs) model the regulatory relationships of transcription factors and genes and have allowed the identification of differentially regulated processes in disease systems. In this perspective, we discuss gene targeting scores, which measure changes in inferred regulatory network interactions, and their use in identifying disease-relevant processes. In addition, we present an example analysis for pancreatic ductal adenocarcinoma (PDAC), demonstrating the power of gene targeting scores to identify differential processes between complex phenotypes, processes that would have been missed by only performing differential expression analysis. This example demonstrates that gene targeting scores are an invaluable addition to gene expression analysis in the characterization of diseases and other complex phenotypes.

## Introduction

A core tenet of molecular biology is that phenotypic differences are reflected through patterns of differential expression of key genes involved in relevant biological processes. Since its inception, whole-genome transcript profiling has been an invaluable tool for exploring these associations and has been used in a range of applications, including identification of clinically relevant molecular subtypes in cancer exhibiting different morbidities and implications for treatment together with characteristic genes associated with these phenotypes (Rouzier et al., 2005; Collisson et al., 2011; Moffitt et al., 2015; Bailey et al., 2016; Kwa et al., 2017; Rodriguez-Salas et al., 2017; Rudin et al., 2019; Sjödahl et al., 2019). Studies have also found that complex patterns of association between genes represented as networks can provide additional insight and that network metrics parameterizing these associations can be used to prioritize and identify crucial disease-related genes (Ramadan et al., 2016; Dimitrakopoulos et al., 2018; Horn et al., 2018; Gumpinger et al., 2020). However, there is growing evidence that the processes regulating the expression of phenotype-associated genes can provide a more holistic picture of drivers of disease and other phenotypes. Gene regulatory networks (GRNs) are often represented as directed bipartite graphs that are used to depict inferred relationships between transcription factors (TFs) and their target genes. GRNs can be characterized by calculating “gene targeting scores,” a network topology measure that captures the complex relationships that a gene has with other TFs and genes and represents the extent to which a gene is targeted in a given system. In this perspective, we will present gene targeting scores, discuss their meaning, and show how this network-based measure provides information about disease systems beyond that found using only differential expression analysis in the investigation and characterization of human disease.

## Gene Regulatory Networks: Characterization of Systems

**FIGURE 1:**
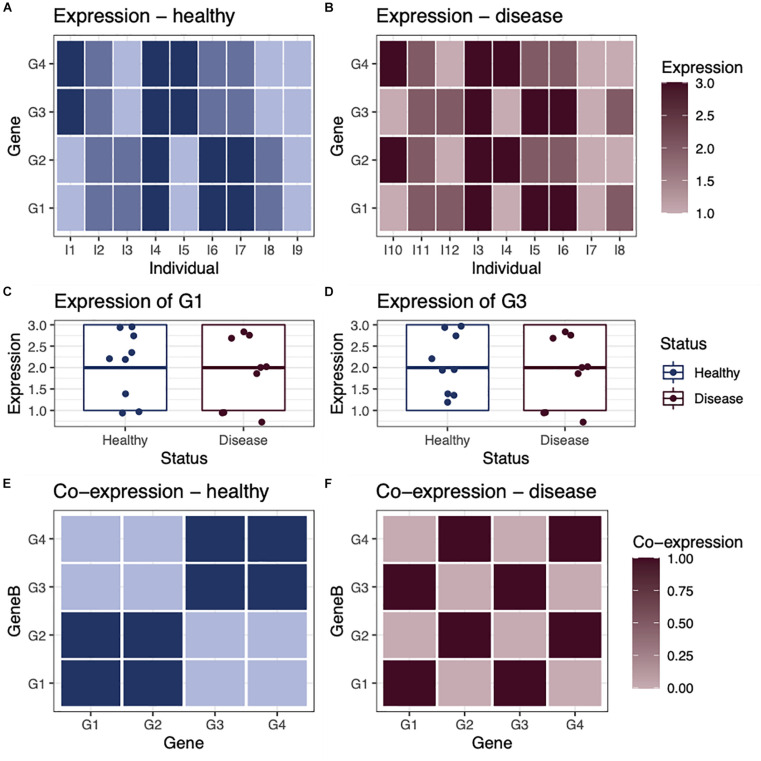
Differential expression vs. differential co-expression. As a toy example, we consider the expression of four genes in **(A)** nine healthy individuals and **(B)** nine individuals with a disease. In this example, none of the genes are differentially expressed, as they have a similar average expression level in both healthy and disease individuals, shown in examples **(C)** gene G1 and **(D)** gene G3. However, when we look at the co-expression between genes within healthy individuals **(E)** and within disease individuals **(F)**, we see that there is obvious differential co-expression between genes in healthy individuals, compared with disease individuals.

Networks are useful tools for representing and analyzing large, complex datasets because they capture information about the *relationships* within a system rather than simply the state of individual components. This is an important distinction, as can be illustrated with a small toy example first described in Glass et al. (2014; Figure 1). In this example, we consider the expression of four genes in nine healthy individuals and nine individuals with a disease (Figures 1A,B). Comparing expression levels between healthy and diseased individuals, we find that none are differentially expressed (Figures 1C,D). However, when looking at the co-expression of these genes in each group of individuals, we see that that the genes are *differentially co-expressed* between groups. For example, in healthy individuals, gene G1 is co-expressed with gene G2 (Figure 1E), whereas in diseased individuals, gene G1 is co-expressed with gene G3 (Figure 1F). This illustrates that differential expression analysis alone may miss important correlations or regulatory relationships that distinguish biological states such as healthy and disease.

This does not mean that gene expression analysis alone is not useful. Differential expression analyses have contributed to many key advances in our understanding of disease. For example, much of our understanding of the complexities of human cancers is derived from large-scale expression profiling of cancer, such as that carried out by The Cancer Genome Atlas (TCGA)^1^, where the expression-based subtypes that have been identified possess distinct clinical characteristics. In pancreatic ductal adenocarcinoma (PDAC), several studies have used expression profiling to identify molecular subtypes (Collisson et al., 2011; Moffitt et al., 2015; Bailey et al., 2016; Rashid et al., 2020; Puleo et al., 2018; Maurer et al., 2019). However, we suggest that a more comprehensive molecular characterization of diseases can be achieved by exploring inferred regulatory network differences and differential gene targeting.

**FIGURE 2:**
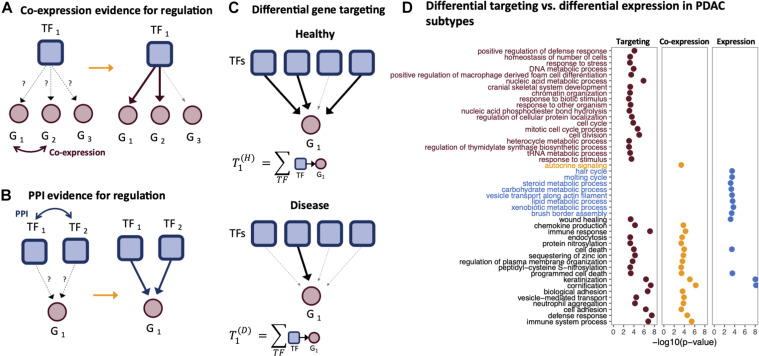
Gene targeting. Gene targeting scores are derived from gene regulatory networks (GRNs) and thus are influenced by the components used to derive the edge weights of a GRN. For example, PANDA GRNs include information regarding the **(A)** co-expression relationships between genes and **(B)** protein–protein interactions between transcription factors (TFs). **(C)** Gene targeting scores are calculated as the sum of the weights across all inbound edges pointing to a gene. **(D)** Gene Ontology (GO) enrichment of ranked differential gene scores comparing the basal-like and classical pancreatic ductal adenocarcinoma (PDAC) subtypes. Genes were ranked by differential targeting (red), differential co-expression (orange), and differential expression (blue).

In 2013, Glass et al. (2013) introduced the PANDA (Passing Attributes between Networks for Data Assimilation) framework for GRN construction. This method takes a data integration approach to GRN construction by using message passing to combine multiple data sources. PANDA predicts regulatory relationships between TFs and genes by considering three main sources of information: (1) a TF–gene network “adjacency matrix” representing an initial guess of which TFs regulate which genes based on the presence/absence of TF motif in the promoter regions of genes, (2) a protein–protein interaction network “co-operativity matrix” that recognizes that many TFs exert their influence through regulatory complexes, and (3) a gene co-expression matrix representing gene–gene relationships initially based on correlation in expression patterns across a set of samples. These three different sources of information are iteratively updated using a message-passing algorithm, using the logic that if two genes are co-expressed, they are more likely to be co-regulated by a similar set of TFs (Figure 2A), and that if two TFs interact, they are more likely to bind promoter regions as a complex and co-regulate the expression of their target genes (Figure 2B). In this process, the TF–gene “edge weights” in the adjacency matrix are updated to reflect the evidence supporting a regulatory interaction; the refinement of edge weights through message passing has been found to improve the prediction accuracy of GRNs, validated through prediction of chromatin immunoprecipitation (ChIP)-seq-derived TF binding.

PANDA has been used to investigate gene regulatory relationships in several disease contexts, including chronic obstructive pulmonary disease (COPD) (Glass et al., 2014), asthma (Qiu et al., 2018), ovarian cancer (Glass et al., 2015), and colorectal cancer (Vargas et al., 2016; Lopes-Ramos et al., 2018). In addition, single-sample versions of PANDA GRNs, derived using a method called LIONESS (Linear Interpolation to Obtain Network Estimates for Single Samples) (Kuijjer et al., 2019b), have been used to study sex-linked differences in colon cancer (Lopes-Ramos et al., 2018) as well sex-related differences in gene regulation (Lopes-Ramos et al., 2020) in tissues from the Genotype-Tissue Expression (GTEx) project (Lonsdale et al., 2013).

## Gene Targeting Score: Identifying Informative Regulatory Processes

The use of GRNs in the analysis of disease relies on analysis of the “gene targeting score,” a numerical score representing the extent to which a gene is targeted by TFs in a given biological context. The gene targeting score is calculated by summing the weights of all inbound regulatory edges for a gene (Figure 2C). Because of the way in which PANDA estimates edge weights, a gene’s targeting score synthesizes multiple lines of evidence—TF motif data, TF–TF interactions, and gene expression correlation. Thus, gene targeting scores are not necessarily correlated with absolute gene expression levels, and consequently, differential targeting is not necessarily correlated with differential gene expression.

Sonawane et al. (2017) used PANDA to construct tissue-specific GRNs for 38 tissues in GTEx and investigated the tissue specificity of TF–gene regulatory relationships. They found many tissue-specific regulatory relationships that would have been missed by using expression information alone. For example, when comparing the tissue-specific regulatory activity of TFs based on gene expression with that deduced using network targeting, they found that TF regulation of tissue-specific function was evident when using gene targeting metrics, but it was largely independent of TF expression level (Sonawane et al., 2017). PANDA analysis also identified unique, tissue-specific targeting patterns in the TF–gene edges and found significant enrichment of tissue-specific regulatory edges targeting tissue-specific expressed genes. This example demonstrates how differences in tissue-specific regulatory relationships between TFs and genes can give rise to the distinct phenotype by altering regulation of key biological processes (Sonawane et al., 2017).

A study of COPD by Glass et al. (2014) found patterns of gene targeting that differed between men and women and offered a possible explanation for higher disease susceptibility of women compared with men. They first compared gene expression between males and females with COPD and found little evidence of differential expression of autosomal genes. Using a resampling approach, they constructed an ensemble of 100 male and 100 female PANDA GRNs. They calculated a targeting score for each gene in each network defined as the sum of all inbound edge weights for the gene. They found several genes that were not differentially expressed but nevertheless had significantly different targeting scores between the sexes. Pre-ranked gene set enrichment analysis based on targeting scores found many processes associated with mitochondrial function that were more highly targeted in females; these processes had previously been implicated in many aspects of COPD and lung disease (Glass et al., 2014). These results suggest that that differential regulation of processes associated with disease may alter disease development and progression in meaningful ways.

Lopes-Ramos et al. (2020) investigated sex differences in gene expression and gene regulation in 29 human tissues by constructing individual-specific networks for each sample in each tissue. Differential edge weights between males and females were identified, and genes were classified as differentially targeted if at least 5% of their inbound edge weights were significantly different between males and females. This allowed genes to be classified as being male-biased if most (>60%) of the inbound differential edges were higher in males, female-biased if most (>60%) of the inbound differential edges were higher in females, and sex-divergent if the number of inbound differential edges was evenly split between being higher in males and higher in females (Lopes-Ramos et al., 2020). Consistent with previous studies, they found little differential gene expression except in breast tissue, with the median number of differentially expressed genes across tissues equal to 64. However, widespread sex-biased targeting was detected in all tissues, with a median number of differentially targeted genes across tissues equal to 169. Interestingly, the sex hormone receptors ESR1, ESR2, and AR were differentially targeted between male and female individuals in several tissues such as the breast, heart, and blood, despite the fact that those hormone receptors were not differentially expressed.

## Specific Example: Pancreatic Ductal Adenocarcinoma Subtypes

PDAC is a lethal disease involving heterogeneous tumors composed of diverse cell types including tumor epithelial cells and components of the tumor microenvironment such as immune cells and fibroblasts. Molecular subtypes of PDAC have been identified through gene expression analysis (Collisson et al., 2011; Moffitt et al., 2015; Bailey et al., 2016; Puleo et al., 2018; Maurer et al., 2019), and the basal-like and classical subtypes first identified by Moffitt et al. have been associated with both prognosis and treatment response (Moffitt et al., 2015; Aung et al., 2018; Rashid et al., 2020; O’Kane et al., 2020). The basal-like subtype is associated with worse median patient survival and resistance to chemotherapy (Moffitt et al., 2015; O’Kane et al., 2020) and has characteristically high expression of keratins and laminins, both structural proteins also associated with the basal subtypes of breast and bladder cancers (Damrauer et al., 2014; McConkey et al., 2014; Weinstein et al., 2014). The classical subtype shows better response to treatment and better overall survival and is marked by increased expression of GATA binding protein 6 (GATA6), a TF involved in cell differentiation.

To identify factors driving these subtypes, we compared differential gene expression and differential GRN gene targeting scores between basal-like and classical subtypes of 150 PDAC tumors using TCGA (Cancer Genome Atlas Research Network, 2017) transcripts per kilobase million (TPM) expression data processed using Recount (Collado-Torres et al., 2017). We used PANDA and LIONESS to construct sample specific GRNs and chose to limit our analysis to those genes with a high standard deviation of logTPMs [sd(logTPMs) > 0.4] across samples. For each gene in each individual tumor, a gene targeting score was calculated as the sum of all inbound edge weights surrounding each gene. Separately, we also calculated a sample-specific co-expression network for each tumor (Kuijjer et al., 2019a) and for each gene in each sample, and we calculated a gene co-expression score equal to the sum of each gene’s co-expression edges surrounding the gene. We used limma (Ritchie et al., 2015) to compare the expression data, the correlation networks, and GRNs between the basal-like and classical subtypes, allowing us to identify differentially expressed genes, differentially co-expressed genes, and differentially targeted genes, respectively.

The three genes found to be most significantly differentially targeted in GRNs, but not differentially expressed, between basal-like and classical subtypes are folate receptor beta (FOLR2), hedgehog interacting protein (HHIP), and the CD209 antigen C-type lectin domain family 4 member L (CD209). FOLR2 encodes the folate receptor 2 protein and is known to be overexpressed in tumor-associated macrophages (Tie et al., 2020). HHIP codes for the hedgehog interacting protein; the hedgehog signaling pathway regulates cell differentiation and proliferation and is activated in several cancers including PDAC (Yang et al., 2010; Honselmann et al., 2015; Gu et al., 2016). CD209 codes for a C-type lectin domain family 4 protein and is a dendritic cell marker. The roles that these play in PDAC have not yet been explored.

Ranking genes according to three different metrics, differential targeting, differential co-expression, and differential expression, we performed ranked gene set enrichment analysis (Eden et al., 2009; Supek et al., 2011) to identify significantly over-represented biological process Gene Ontology (GO) terms. Both the differential targeting analysis and differential expression analysis identified keratinization, cornification, cell death, and wound healing as differentiating basal-like and classical samples. However, several immune-related processes, epigenetic, and cell cycle processes found by differential targeting analysis were missed using differential expression alone (Figure 2). Functional enrichment using co-expression scores to rank genes identified some processes similar to those found using differential targeting but missed several important pathways related to cell cycle and other processes, such as chromatin organization.

The identification of keratinization as enriched in differentially expressed genes is consistent with previous studies that identified genes encoding keratins and laminins as biomarkers for basal-like tumors (Moffitt et al., 2015, O’Kane et al., 2020). The fact that both differential expression and differential targeting identified keratinization and cell adhesion as biological processes distinguishing PDAC subtypes serves as an internal consistency check. Differential targeting alone identified processes related to the immune system and speaks to the importance of the tumor microenvironment, which is known to influence PDAC prognosis and drug response. A high degree of tumor-associated macrophage infiltration has been linked to lower survival (Karamitopoulou, 2019), which is a known hallmark of the basal-like subtype, and it is possible that the differential targeting analysis is detecting cross-talk between the tumor and the tumor microenvironment. The differential targeting of epigenetic functions between subtypes is consistent with reports that the basal-like and classical subtypes have distinct epigenetic landscapes (Lomberk et al., 2018).

This analysis of PDAC subtypes, although abbreviated, demonstrates the power of using GRN inference and gene targeting score analysis to identify regulatory processes that characterize distinct phenotypes—including processes that are distinct from those that are associated with patterns of gene expression. The biologically relevant differences we see in targeting but not expression or co-expression suggest that regulatory control, even if not activated, is important in defining health and disease.

## Discussion

There is growing experimental evidence of the importance of complex regulatory processes in distinguishing phenotypes in health and disease. For example, the Wilms tumor-1 (WT1) TF is a master regulator that targets several essential genes in kidney podocyte cells. Ettou et al. (2020) investigated WT1-based gene regulation during podocyte injury and found that WT1 maintained open chromatin in the regions of its target genes but that the expression level of WT1 was not universally associated with the intensity of its binding. They also found that WT1 could cause either an increase or a decrease in the expression of its target genes. The role of complex regulatory processes is further illustrated by the work of Carnesecchi et al. (2020), who investigated how a single TF could regulate different developmental programs in various cell lineages. They showed that the Ubx TF forms different complexes with distinct binding partners in various cell lineages despite the fact that most of the interaction partners showed no differential expression across the lineages.

Taken together, the results reported by Ettou and Carnesecchi illustrate the complexity of regulatory processes and the importance that regulatory targeting plays in defining phenotype, even in instances in which a key regulator does not itself substantially change in its expression levels. Their results also point to the importance of modeling both “direct” and “indirect” regulation of genes by TFs and the complexes they form. Among the methods for GRN inference, PANDA (and by extension, PANDA+LIONESS) is singular in considering interactions between TF proteins in its model. PANDA’s integrative approach using TF–TF interactions, predicted TF–gene regulatory relationships, and gene co-expression data, refines the inputs to optimize agreement between them; the resulting networks provide unique insight into regulatory processes that are linked to phenotypes.

The work summarized in this perspective demonstrates the value of the gene targeting score as a metric for assessing the drivers of phenotypic differences. Gene targeting scores not only capture structural characteristics of regulatory networks but also allow for the identification of processes that may be activated in response to appropriate stimuli and in this way help to define phenotypes and disease subtypes. For example, Lopes-Ramos et al. (2018) performed gene targeting analysis on gene expression data from colon cancer tumor samples and discovered differences between males and females in the regulation of genes involved drug metabolism, suggesting that male and female tumor cells are programmed to respond differently. They found that genes in drug metabolism pathways, particularly those acting through cytochrome P450, had higher targeting scores in female networks than male networks. Furthermore, higher targeting of the drug metabolism pathways was found to correlate with patient survival, indicating a mechanism for sex-divergent response to chemotherapy in colon cancer.

Our application of PANDA and LIONESS in comparing PDAC subtypes demonstrates the value of the GRN-based approach and of using network-based metrics such as gene targeting to characterize properties of biological systems. We constructed sample-specific GRNs for 150 PDAC tumors and used gene targeting to compare the topologies of networks derived from basal-like and classical tumor subtypes. We found that differential targeting analysis identified compelling differences between the two subtypes in the regulation of processes related to cell cycle, immune, and epigenetic functions, none of which were seen in a standard differential expression analysis. Given that PDAC tumors are known to exhibit immune infiltration, and that the subtypes differ in both their epigenetic landscapes and patient survival, our identification of relevant processes illustrates how GRN-based methods can provide important and relevant biological insights into disease-associated processes beyond what is seen using other analytical methods.

PANDA and LIONESS software for GRN analyses and identification of differential targeting are freely available as open-source tools with extensive documentation (netzoo.github.io) and can easily be implemented in most analytical workflows. We hope that this review motivates the broader use and appreciation of gene targeting analysis.

## Data Availability Statement

Publicly available data analyzed in this study were obtained from recount2 (https://jhubiostatistics.shinyapps.io/recount/). We provide an interactive Jupyter notebook hosted in netbooks (netbooks.networkmedicine.org) to reproduce the differential targeting analysis and the PDAC gene regulatory networks are available in GRAND database (https://grand.networkmedicine.org/cancers).

## Author Contributions

DW drafted the manuscript and performed the analysis. All authors discussed, planned, reviewed and edited the manuscript.

## Conflict of Interest

The authors declare that the research was conducted in the absence of any commercial or financial relationships that could be construed as a potential conflict of interest.

## Footnotes

1 https://www.cancer.gov/tcga
